# Scabrous is distributed via signaling filopodia to modulate Notch response during bristle patterning in Drosophila

**DOI:** 10.1371/journal.pone.0291409

**Published:** 2023-09-20

**Authors:** Adam Presser, Olivia Freund, Theodora Hassapelis, Ginger Hunter

**Affiliations:** Department of Biology, Clarkson University, Potsdam, New York, United States of America; University of Rome, ITALY

## Abstract

During development, cells in tissues must be patterned correctly in order to support tissue function and shape. The sensory bristles of the peripheral nervous system on the thorax of Drosophila melanogaster self-organizes from a unpatterned epithelial tissue to a regular spot pattern during pupal stages. Wild type patterning requires Notch-mediated lateral inhibition. Scabrous is a protein that can bind to and modify Notch receptor activity. Scabrous can be secreted, but it is also known to be localized to basal signaling filopodia, or cytonemes, that play a role in long-range Notch signaling. Here we show that Scabrous is primarily distributed basally, within the range of signaling filopodia extension. We show that filamentous actin dynamics are required for the distribution of Scabrous protein during sensory bristle patterning stages. We show that the Notch response of epithelial cells is sensitive to the level of Scabrous protein being expressed by the sensory bristle precursor cell. Our findings at the cell-level suggest a model for how epithelial cells engaged in lateral inhibition at a distance are sensitive local levels of Scabrous protein.

## Introduction

The timely and ordered patterning of tissues during development contributes to overall tissue homeostasis and animal behavior. The array of microchaete sensory bristles on the dorsal thorax of the fruit fly Drosophila melanogaster is a well-studied example of a self-organizing, repeating spot pattern driven by Notch-mediated lateral inhibition [1–3]. The thoracic epithelia is initially unpatterned, and most cells are able to commit to either an epithelial or sensory bristle precursor cell fate. Over several hours, bristle precursor cells are specified by low levels of activated Notch and increased expression of proneural genes. Bristle precursor cells engage in lateral inhibition with neighboring epithelial cells. A feature of this system is pattern refinement [4, 5], where cells in the patterning tissue are able to flexibly adjust their cell fate during patterning in order to ensure the correct spacing between bristle precursors. For example, if a bristle precursor cell is ablated, a nearby uncommitted epithelial cell can take its place [4, 6]. The ability to switch fates is a property of most epithelial cells in the notum and is linked to Notch signaling status and G2-exit [6, 7]. The flexibility of cells fates in the tissue helps to ensure a robust sensory organ pattern. Failure to correctly organize the thoracic sensory bristles can lead to changes in fly behavioral responses [7–9]. Furthermore the spatial distribution of sensory bristles is under selective pressure as Drosophila species exhibit a variety of bristle densities and organizations [10]. Therefore, it is important to understand the mechanisms that result in successful tissue patterning.

Theoretical and experimental data demonstrate that molecular modifiers of Notch signaling and cell morphology play roles in the density of the final bristle pattern [4, 11, 12]. Models clearly support a characteristic length for the lateral inhibition signal beyond adjacent cells, which could be achieved by active cell processes and by diffusible signals [13]. Dynamic actin-based signaling filopodia, or cytonemes, have been shown to play a role in the Notch response of epithelial cells during patterning stages [4, 14, 15]. Both Notch receptor and Delta ligand have been shown to localize along the length of signaling filopodia [15–17], similar to cytoneme-mediated Notch signaling in other tissues [18, 19]. Filopodia structures extend from the basal surface of all notum epithelial cells and permit cells that are ~2–3 cell diameters away from each other to contact and therefore engage in Notch signaling. Direct evidence of Notch activation along signaling filopodia has not been shown. However, there is genetic evidence that perturbation of actin regulators, and therefore signaling filopodia formation and activity, leads to defects in bristle patterning consistent with mathematical models [4, 14–16].

An alternative to active cell processes extending the range of Notch signaling would be the presence of a diffusible regulator of Notch signaling. Scabrous is a fibrinogen-like protein that binds to Notch receptors and plays a role in the formation of a well-spaced array of sensory bristles in the dorsal thorax [17, 20]. Loss of Scabrous expression leads to an increase in bristle density, although how this is achieved is unclear. Scabrous can contribute to the stability of Notch receptor on the cell surface [20], however, it can also block Delta-Notch interactions [21]. There is evidence that Scabrous is a secreted and diffusible Notch regulator, expressed primarily by Notch-inactive, Delta-expressing bristle precursor cells [22–24]. Other studies indicate that Scabrous is carried via long, actin-rich signaling filopodia [7, 25], the activity of which are also implicated in establishing bristle density as discussed above [4, 15]. Signaling filopodia formation and dynamics are Scabrous independent [17] but both are upregulated in cells that express high levels of Delta ligand [16, 26].

Here we investigate the role of Scabrous in long-range Notch signaling during thoracic bristle patterning. Using genetic and pharmacological strategies, we have found that Scabrous protein is primarily localized to the basal surface of the patterning epithelium, where its distribution is actin-dependent. We further show that loss of the Scabrous gradient leads to disruption of the Notch response in epithelial cells. We propose that Scabrous helps to coordinate pattern refinement by modifying Notch signaling response in epithelial cells that are distant from Delta-expressing bristle precursor cells.

## Results and discussion

### 1. Scabrous distribution in vivo

**Fig 1:**
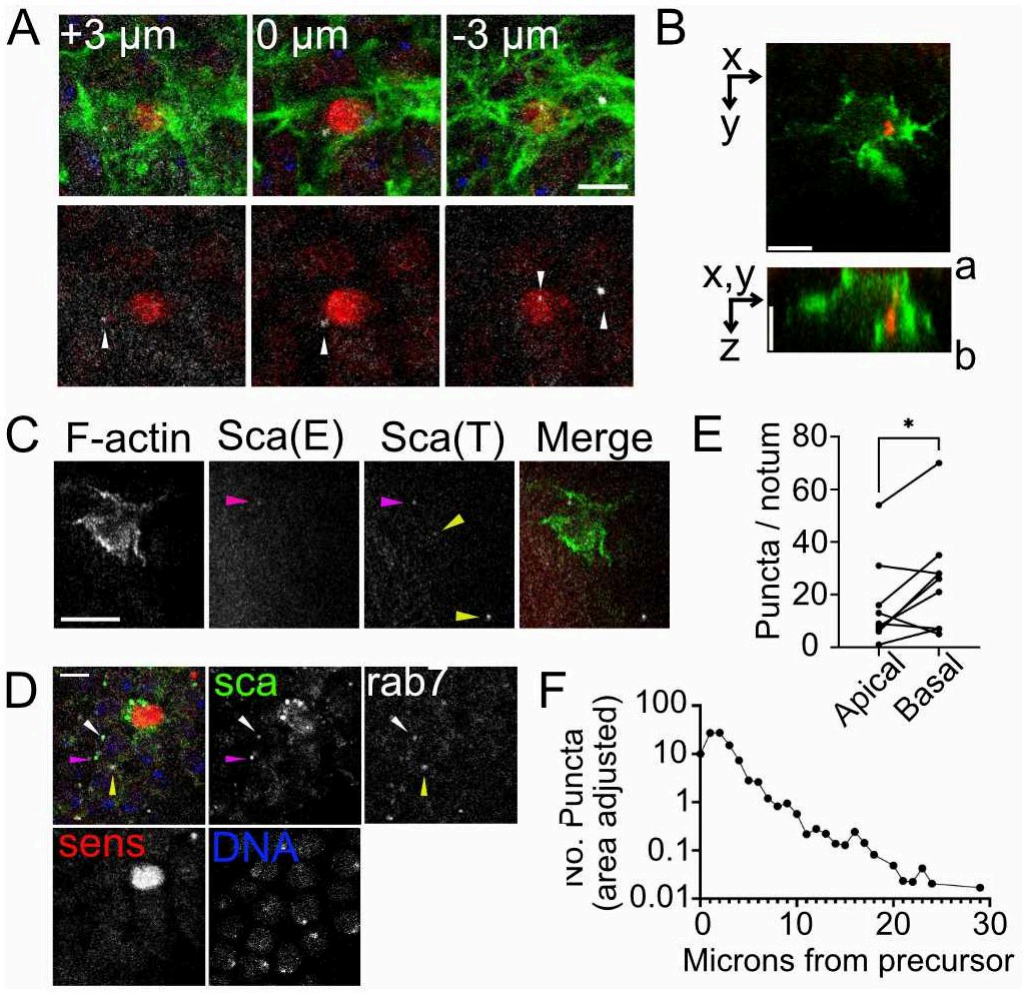
Spatial distribution of Scabrous protein during bristle patterning stages. (A) Example of apicobasal distribution of Scabrous protein (arrowheads, white puncta, anti-Scabrous) in the notum, with bristle precursor cell marked by red nuclei (anti-senseless). Phalloidin is used to stain filamentous actin (green). Scale bar, 5 μm. Apical (+ μm) and basal (- μm) are relative to the middle of the sensless positive nuclei (widest diameter). Genotype: w^1118^. (B) Apicobasal distribution of Scabrous protein (red) in a bristle precursor cell labeled with anti-GFP (green). a, apical; b, basal. Scale bars, 5 μm. Genotype: neur-GAL4, UAS-GMCA/+. (C) Anti-scabrous staining for extracellular only (Sca-E) or total (Sca-T) Scabrous protein near a bristle precursor cell (green in merge). Pink arrowhead = double positive Sca puncta, indicative of extracellular protein. Yellow arrowheads = single positive Sca puncta, stained after permeabilization. Genotype: neur-GAL4, UAS-GMCA/+. Scale bar, 10 μm. (D) Demonstration of Scabrous and Rab7 colocalization patterns. Sca = scabrous, anti-GFP staining; sens = senseless staining to mark bristle cell precursor nuclei. DNA = DAPI stain to mark all nuclei. White arrowhead = puncta double positive for anti-GFP and anti-rab7. Pink arrowhead = puncta single positive for anti-GFP. Yellow arrowhead = puncta single positive for rab7. Note most puncta closely associated with senseless nuclei are single positive for anti-GFP. Genotype: scabrous^GSFTF^. Scale bar, 5 μm. (E) Pairwise quantification of Scabrous puncta at apical or basal z-planes for n = 9 nota. *, p = 0.04, r = 0.83 by paired student’s t-test. (C) XY distribution of Scabrous puncta relative to the center of bristle precursor cells (0 μm). The number of puncta are area adjusted, across n = 9 nota. See also S1 Fig and S1 Movie.

In order to determine the contribution of Scabrous to long-range Notch signaling, we first determined the spatial distribution of endogenous Scabrous protein in the patterning notum (Fig 1A and 1B). We stained for endogenous Scabrous protein in nota at bristle patterning stages (~14 hours after pupariation, hAP). We find that 98.6% of observed bristle precursor cells (n = 73 cells, across 5 pupae) contain at least one Scabrous positive puncta. Most bristle precursor cells contain multiple Scabrous puncta (4.2 ± 1.8 puncta per cell, mean ± SD, n = 73 cells). Endogenously expressed Scabrous protein is primarily localized to puncta in bristle precursor cells (arrowheads, Fig 1A and 1B), which likely represents localization to vesicles, as previously described [17]. In order to determine whether the puncta we observe are intracellular or extracellular, we stained for Scabrous pre-and post-permeabilization (Fig 1C). Double positive puncta (see methods) represent extracellular (E) Scabrous. Only 12.9% of puncta observed (n = 31 puncta across 23 cells) stained double positive, with the majority of Scabrous only staining post-permeabilization (T, total Scabrous: 87.1%). Furthermore, extracellular Scabrous was almost always closely associated with the bristle precursor cell (Fig 1C). To further investigate the distribution of Scabrous in bristle precursor cells and their epithelial cell neighbors, we co-stained tissues expressing endogenously tagged Scabrous^GFP^ with anti-Rab7 antibody which labels late endosomes [27]. Double positive puncta for GFP and anti-Rab7 would represent Scabrous protein that had been endocytosed and are intracellular. Since bristle precursor cells (marked with anti-senseless) are known to express and distribute Scabrous, we expected the bulk of Scabrous^GFP^ in bristle precursor cells would not be associated with endosomes. We find that for Scabrous^GFP^ puncta closely associated with senseless nuclei (n = 86), 19.8% are double positive for Rab7 and GFP. For local puncta (< 15 μm radius from center of senseless nuclei) that are not associated with the senseless nuclei (n = 129), we find that 41.1% are double positive for Rab7 and GFP (white arrowhead in Fig 1D). 58.9% of local Scabrous puncta are not associated with Rab7 (pink arrowhead in Fig 1D). We also observe puncta that stain only for Rab7 but not GFP (yellow arrowhead in Fig 1D). Since our permeabilization experiment suggests that most Scabrous is intracellular, the presence of local Scabrous puncta that do not co-stain for Rab7 may represent puncta that are localized in another vesicular compartment, or Scabrous within signaling filopodia at a distance from the bristle precursor cell. Due to the limitations of the Rab7 experiment, we were not able to co-stain with phalloidin to visualize total filamentous actin.

We next quantified the apical-basal distribution of Scabrous protein. We find that Scabrous puncta distribution is skewed towards the basal side of the tissue (Fig 1E, S1 Fig). Overall, 57.3% of observed bristle precursor cells (n = 73 cells) have at least one Scabrous puncta on the basal side (1.0 ± 0.9 puncta per cell, mean ± SD) and 24.0% of observed bristle precursor cells have any Scabrous puncta on the apical side (0.4 ± 0.8 puncta per cell, mean ± SD). We then determined the overall distribution of Scabrous puncta relative to the nearest bristle precursor cell nuclei. Scabrous puncta can be observed as far as 2–3 cell diameters away (~20–30 microns, Fig 1F), although most are localized within 10 microns of the bristle cell nuclei. Overall, 30.7% of bristle precursor cells had puncta located between 10 and 30 microns away (0.5 ± 0.9 puncta per cell, mean ± SD). Interestingly, the average length of signaling filopodia extending from the basal surface of bristle precursor cells is ~10 μm [15]. Therefore, the XY distribution of Scabrous could be consistent with either active or passive processes.

### 2. Scabrous distribution is dependent on filamentous actin

**Fig 2:**
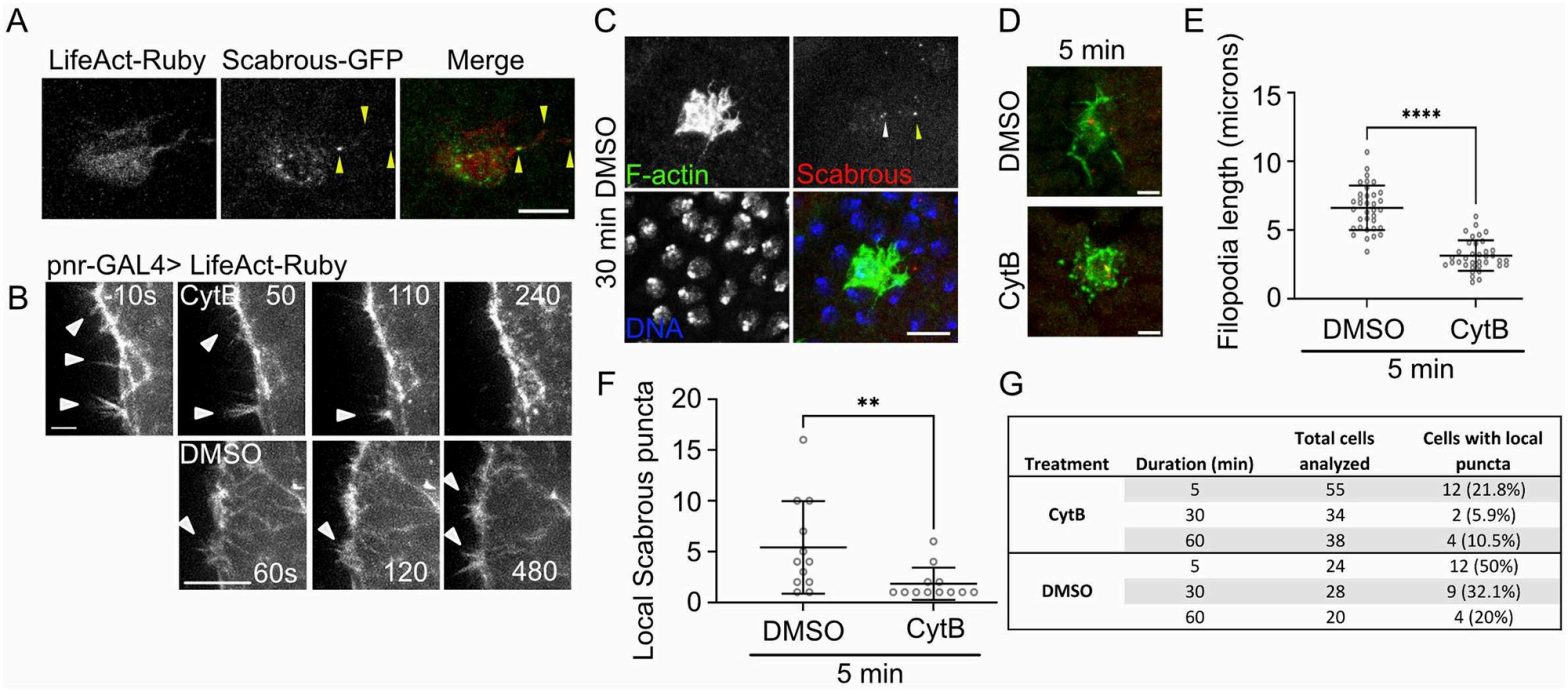
Actin dynamics are required for the local distribution of Scabrous. (A) Bristle precursor cell in vivo co-expressing LifeAct-Ruby to label cell shape and Scabrous^GFP^. Pupae is ~14 hAP. Yellow arrowheads = Scabrous-GFP localized to signaling filopodia. Genotype: UAS-LifeAct-Ruby/UAS-sca^GFP^; neur-GAL4/+. Scale bar, 10 μm. (B) Timelapse panels of nota explants expressing the filamentous actin marker LifeAct-Ruby and treated with either DMSO or Cytochalasin B. Time in seconds. Either treatment was added at time = 0 seconds. Arrowheads point to individual signaling filopodia on the basal surface. Scale bars, 4 μm (CytB) and 10 μm (DMSO). Genotype: UAS-LifeAct-Ruby/+; pnr-GAL4/+. (C) Immunofluorescence image of a sensory bristle precursor cell from a nota under control conditions for 30 minutes prior to fixation. Signaling filopodia are still visible in F-actin panel. White arrow in Scabrous panel points to intracellular Scabrous. Yellow arrow in Scabrous panel points to local Scabrous. Scale bar, 10 μm. Genotype: neur-GAL4, UAS-GMCA/+. (D) Examples cell morphologies upon DMSO control or Cytochalasin B treatments for 5 minutes. Green, anti-GFP (GFP-actin binding domain of moesin labeling F-actin). Red, anti-Scabrous. Scale bar, 5 μm. Genotype: neur-GAL4, UAS-GMCA/+. (E) Quantification of signaling filopodia length after 5 minutes of treatment with either DMSO (n = 35 filopodia across 17 cells) or Cytochalasin B (n = 36 filopodia across 29 cells). ****, p<0.0001 by student’s t-test. Mean ± SD shown. (F) Quantification of local Scabrous puncta within a 15 μm radius of a bristle precursor cell in nota treated with either DMSO (n = 12 cells with local puncta), or cytochalasin B (n = 12 cells with local puncta) for 5 minutes prior to fixation. A minimum of 5 nota were dissected and treated for each time point and condition. **, p = 0.007 by Mann-Whitney test. Mean ± SD shown. (G) Summary of Cytochalasin B experiment. See also S2 Fig.

We and others have observed that Scabrous is distributed both apically and basally in patterning tissues [17, 24]. In a previous study, Scabrous was shown to localize along the length of basal signaling filopodia in bristle precursor cells [7]. We also show that GFP-tagged Scabrous can localize along the lengths of signaling filopodia in live bristle precursor cells (Fig 2A). It is currently unknown if Scabrous is distributed via these signaling filopodia, through a diffusion-based process, or both. There is no experimental evidence that Scabrous leaves the cell body through apical secretion or basally via filopodia. Importantly, while both Scabrous and signaling filopodia are upregulated in cells that express Delta, the formation of signaling filopodia is not Scabrous dependent [16, 17]. Therefore we next asked whether the distribution of Scabrous in vivo is dependent on the activity of actin-based signaling filopodia.

Since signaling filopodia are actin-dependent structures, we used the filamentous actin (F-actin) inhibitor Cytochalasin B to acutely disrupt actin during patterning stages [28]. Previously, we had adapted an ex vivo technique for the pharmacological perturbation of nota [15, 29]. We found that treatment with Cytochalasin B leads to the rapid disassembly of signaling filopodia in notum explants, compared to DMSO controls (Fig 2B). F-actin disruption, visualized by loss of LifeAct-Ruby containing cell protrusions, was apparent by < 60 seconds after addition of Cytochalasin B. We hypothesized that if signaling filopodia are essential to Scabrous distribution, then loss of filamentous actin dynamics would lead to an decrease in the number of Scabrous puncta at a distance from bristle precursor cells. We exposed dissected nota to Cytochalasin B or DMSO (Fig 2C–2G). We then performed immunostaining at designated time points in order to determine when and how the distribution of Scabrous changed in response to F-actin disruption. We find that long-term incubations with DMSO alone do not disrupt the formation of F-actin based signaling filopodia (Fig 2C). For these experiments, we used the neur-GAL4, UAS-GMCA [4, 14, 15] genetic background that labels bristle precursor cells only with a GFP-tagged actin binding domain of Moesin (GMCA; [30]). We stained total filamentous actin using fluorescent phalloidin to verify the apical-basal location of local Scabrous puncta not associated with bristle precursor cells (S2A Fig).

Treatment of dissected nota with Cytochalasin B leads to disruption of the F-actin cytoskeleton by the first time point observed (5 minutes), indicated by disruption of GMCA localization in bristle precursor cells (Fig 2D) and loss of phalloidin staining across the tissue (S2A Fig). Bristle precursor cells in tissues exposed to control conditions for 5 minutes had more signaling filopodia (5.3 ± 2.1 filopodia per cell, mean ± SD, n = 11 cells) than those exposed to cytohalasin B for the same amount of time (2.1 ± 1.5 filopodia per cell, mean ± SD, n = 14 cells) (Fig 2C). We find that 5 minutes of cytochalasin B treatment is not sufficient to disrupt the total number of Scabrous puncta per bristle precursor cell (5.9 ± 2.5 puncta in control and 4.6 ± 1.8 puncta in cytochalasin B treated, mean ± SD, n.s. by student’s t-test). However, fewer Scabrous puncta are found on basal filopodia following 5 minutes of cytochalasin B treatment (0.45 ± 0.52 puncta in control filopodia and 0 puncta in cytochalasin B treated filopodia, mean ± SD, 0.009. by student’s t-test). We could not analyze conditions beyond 5 minutes of control or cytochalasin B treatment as cells treated at longer time points rarely exhibit basal signaling filopodia (1.0 ± 1.1 per cell at 30 minutes treatment and 0.4 ± 0.6 per cell at 60 minutes treatment, mean ± SD, n = 30 cells each).

To quantify Scabrous distribution under disrupted or control conditions, we count the number of Scabrous-positive puncta within signaling filopodia range of a bristle precursor cell. The average maximum length of signaling filopodia extending from bristle precursor cells is ~10 μm [4, 15], and the average diameter of a bristle precursor cell prior to cell division is ~10 μm. Therefore, we performed our analysis within a 15 μm radius of each bristle precursor cell. Scabrous-positive puncta that did not colocalize with anti-GFP were considered local to the bristle precursor cells, and Scabrous-positive puncta that colocalized with anti-GFP were considered intracellular to the bristle precursor cells (Fig 2C, arrowheads). Since bristle precursor cells extend actin-based protrusions from both lateral and basal surfaces [14] that could potentially carry Scabrous, we focused on locally distributed Scabrous puncta that did not colocalize with anti-GFP, excluding puncta on the apical plane of the tissue. We found that the number of local Scabrous puncta was decreased after cytochalasin B treatment compared to controls (Fig 2F–2G). Overall, fewer cytochalasin B treated cells exhibited any local Scabrous puncta at observed time points (5, 30 and 60 minutes) compared to DMSO controls (Fig 2G). At the 5 minute time point, cytochalasin B treated cells that did exhibit local Scabrous had fewer puncta than DMSO controls (1.8 ± 1.6 puncta (n = 12) and 5.4 ± 4.6 puncta (n = 12) respectively, p = 0.007 by Mann-Whitney test) (Fig 2F). There were not enough cells with local puncta at later time points under cytochalasin B conditions for analysis (Fig 2G). This finding supports a role for filamentous actin in the distribution of Scabrous. It remains to be seen if the effect of Scabrous on Notch signaling requires it to be transferred from the signaling to the receiving cell, similar to what has been observed for Sonic Hedgehog signaling along cytonemes [31].

Several steps in the secretory pathway and exocytosis require the activity of actin cytoskeleton [32, 33]. We would expect that treatment of tissues with cytochalasin B would also potentially disrupt the secretion of diffusible Scabrous [34]. We predicted we would see increased intracellular localization of Scabrous if its secretion were blocked by actin disruption. At 5 and 30 minutes of cytochalasin B treatment, we observe no differences in the number of intracellular Scabrous puncta relative to controls (S2B and S2C Fig). We observed increased levels of intracellular Scabrous puncta at lateral and basal planes when tissues are treated with cytochalasin B for longer periods of time (60 minutes, S1C Fig). This result indicates that disruption of the actin cytoskeleton is having an effect on the retention of Scabrous in the bristle precursor cell at longer timescales. We also observe an overall trend in both DMSO and Cytochalasin B treated tissues of decreased Scabrous puncta over time (S2B and S2C Fig), with controls decreasing to a greater extent than drug treated tissues. It is unclear why this might be the case, whether the cells are affected by the protocol or if they are downregulating Scabrous as part of the normal differentiation process. In vivo, bristle precursor cells commit to the bristle lineage and begin a series of asymmetric cell divisions, approximately 16–18 hAP. There is some evidence that *scabrous* is expressed in all subsequent bristle lineage cells [35], although the levels and distribution of Scabrous protein at that point is unclear. Importantly, at these timescales (60 minutes) we do not observe evidence of increased cell death by nuclear morphology and staining with DAPI. Altogether, our data suggest that the differences we see in the local distribution of Scabrous at shorter timescales requires actin dynamics.

### 3. Changes in Scabrous expression levels leads to changes in Notch signaling

Scabrous is known to directly bind to and modulate Notch signaling [20, 36], although its effect on Notch response is unclear. Expression of Scabrous in wing or eye disc clones leads to inactivation of Notch signaling [20, 21]. In the notum, the Scabrous null phenotype resembles the Notch or Delta hypomorph phenotypes of increased bristles [17], suggesting that it positively regulates Notch activation in the notum. A key difference between these phenotypes is bristle spacing: adjacent bristles are not observed in Scabrous mutant tissues, whereas they are common in Delta or Notch mutant tissues [17, 37]. Scabrous could exclusively play a role in the Notch activity of epithelial cells distant from the bristle precursor. Alternatively, there could be mechanisms that make epithelial cells adjacent to the bristle precursor less sensitive to Scabrous activity. Based on these studies [17, 37], we hypothesized that decreased Scabrous expression would lead to decreased Notch response in cells that are distant from the source of Delta signal. This could account for the increased number of Notch-inactive cells, and increase in bristle density, in Scabrous mutant or knockdown tissues.

**Fig 3:**
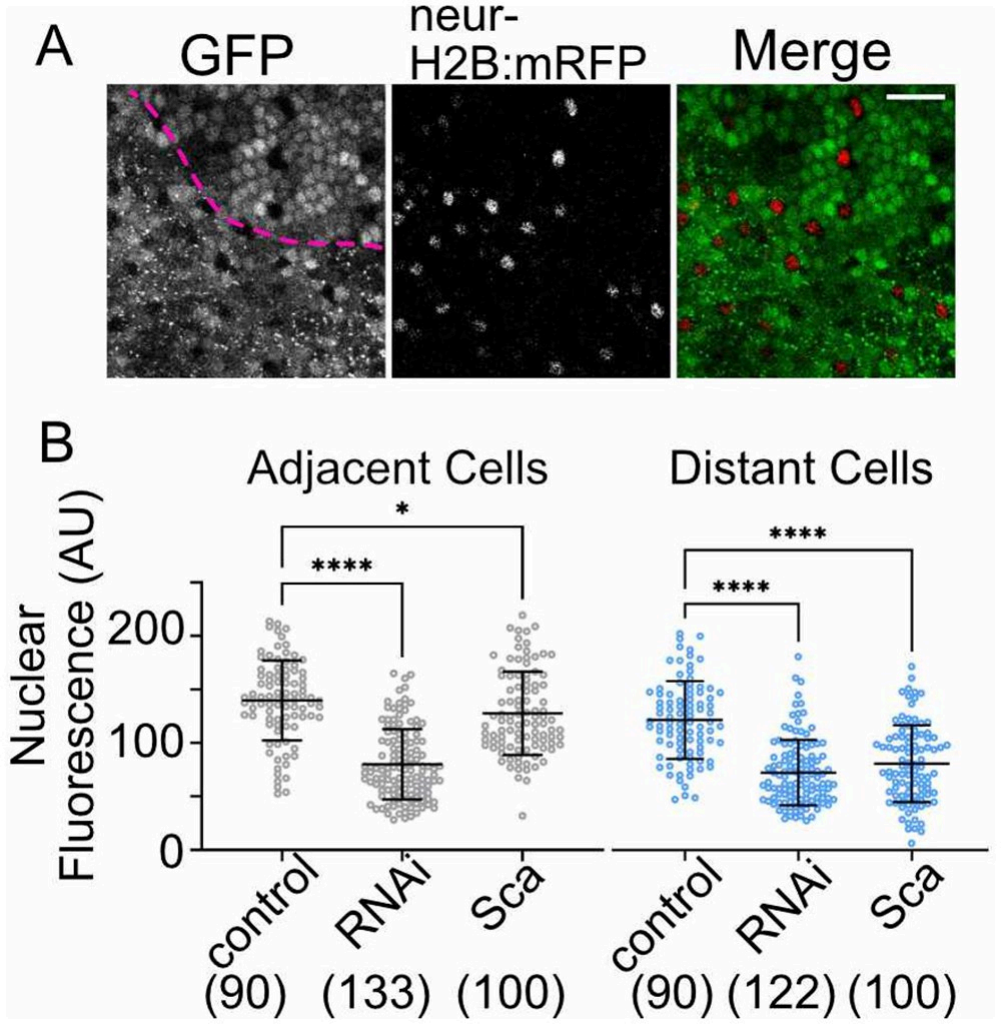
Local Scabrous expression levels are essential for robust Notch response. (A) Example of a live pupal notum expressing UAS-Scabrous GFP in the pannier-GAL4 domain. Lateral edge of the pannier domain is marked by the dashed pink line. All Notch activated cells express the NsfGFP transcriptional reporter, which localizes GFP signal to the nucleus. Bristle precursor cells do no express NsfGFP but do express the neuralized reporter, neur-H2B:mRFP, marking their nuclei. Scale bar, 25 μm. (B) Nuclear GFP fluorescence was measured for cells adjacent to a bristle precursor cell or distant to a bristle precursor cell (+1 nuclei removed) in 14 hAP nota expressing NsfGFP. Tissues were either expressing lower amounts of Scabrous (RNAi = pannier-GAL4 > UAS-Scabrous RNAi), control levels of Scabrous (control = pannier GAL4 > UAS-white RNAi), or elevated levels of Scabrous (Sca = pannier GAL4 > UAS-Scabrous GFP). ****, p<0.0001 and *, p = 0.04 by ordinary one-way ANOVA with multiple comparisons. (n) = number of nuclei measured. A minimum of 3 animals were imaged for each genotype. See also S3 Fig.

To test our hypothesis, we modulated the level of Scabrous expression in nota that expressed a transcriptional reporter of Notch signaling, NsfGFP [6]. Here, nuclear GFP is expressed downstream of a minimal Notch responsive promoter, such that increased levels of nuclear GFP fluorescence is associated with higher Notch signaling activity. We used RNAi to decrease the level of Scabrous throughout the notum. We then quantified the nuclear NsfGFP levels in two populations of epithelial cells: (1) adjacent cells, which are those that share a large cell-cell interface with the Delta-expressing bristle precursor cell; (2) distant cells, which are those that are at least one nuclei removed from the bristle precursor cell. Cells more than one nuclei removed from a bristle precursor cell are rare in these modified tissues, as loss of Scabrous phenotype is the increase the density of bristle precursor cells. Both adjacent and distant epithelial cells are capable of contacting nearby bristle precursor cells via signaling filopodia. However, distant epithelial cells may only use signaling filopodia to engage in contact-mediated Notch signaling with the bristle precursor cells. At this time, we are unaware of any experimental approach that would allow us to directly link filopodia engagement and signaling with Notch response. However, we can investigate the impact that Scabrous has on filopodia-mediated signaling by observing the effect of manipulating Scabrous levels on epithelial cells that are distant from bristle precursor cells. We measured the nuclear NsfGFP fluorescence levels in 14 hAP nota expressing UAS-scabrous RNAi to decrease Scabrous expression, compared to UAS-white RNAi expressing control, both under the pannier-GAL4 driver that expresses throughout the central notum (Fig 3A and S3 Fig).

In control tissues, epithelial cells adjacent to bristle precursor cells have a higher level of nuclear NsfGFP reporter than distant cells [6]. This has been interpreted to be due to increased surface contact area between adjacent epithelial cells and bristle precursor cells leading to increased Notch-Delta engagement and activation [38]. When Scabrous levels are decreased via the expression of Scabrous RNAi, we observe that nuclear NsfGFP reporter levels decrease in both adjacent and distant epithelial cells (Fig 3B, RNAi). This suggests that Scabrous acts as a positive regulator of Notch activity in the notum. We also find that the mean nuclear NsfGFP fluorescence for adjacent and distant cells in Scabrous RNAi expressing nota is not significantly different (student’s t-test). In the context of previously published results showing that Scabrous mutants have decreased spacing between bristles, but never adjacent bristles, suggests either that (1) the level of Notch activation in adjacent epithelial cell neighbors surpasses a threshold for acquiring the epithelial cell fate or (2) a low, but sufficient, level of Scabrous still being expressed and promoting Notch activation. Due to the short predicted half-life of Scabrous protein [39] as well as the ability of pnr-GAL4> UAS-Scabrous RNAi nota to display a Scabrous mutant phenotype of increased bristle density, we favor the threshold model.

Previously it was shown that overexpression of Scabrous throughout a patterning tissue is unable to rescue spacing phenotypes [17, 39]. It was proposed that this is because local differences in Scabrous levels play a key role in patterning and lateral inhibition. We found that when Scabrous was overexpressed throughout the notum, nuclear NsfGFP reporter levels were decreased relative to controls (Fig 3B, Sca). Adjacent epithelial cells in Scabrous overexpressing tissues exhibit a Notch activity that is only slightly decreased relative to controls. Distant epithelial cells in Scabrous overexpressing tissues exhibit strong decreases in Notch activity relative to controls, comparable to distant cells in Scabrous RNAi expressing tissues. We also find that the mean nuclear NsfGFP fluorescence for adjacent and distant cells in Scabrous over-expressing nota is significantly different (p<0.0001, student’s t-test). Together with the observation that the difference between Notch response in adjacent and distant cells is lost in Scabrous RNAi expressing tissues, this indicates that Scabrous plays a role spatial organization of Notch responsive cells. This is important because previous studies have shown that differential Notch responses in adjacent and distant epithelial cells lead to changes in the timing of G2-exit, which impacts the overall tissue pattern [6]. Our results are similar to those observed in the wing disc [21], where ectopic expression of Scabrous protein led to the downregulation of genes downstream of Notch activation. Our results give cell level detail for earlier observations that loss of Scabrous expression and uniform Scabrous overexpression both lead to spacing phenotypes [17, 39]. The decreased Notch response in distant epithelial cells for both RNAi and overexpression conditions may be sufficiently low as to allow cell fate switching, where these distant epithelial cells become bristle precursors.

It remains unclear as to why epithelial cells adjacent to bristle precursors are less affected by overexpression of Scabrous compared to epithelial cells distant to bristle precursors. A major difference between the two epithelial cell types is surface area of contact with the nearest Delta-expressing bristle precursor cell. Assuming both epithelial cell types express Notch receptor at similar levels, contact area differences may have significant impact on the relative numbers of free or engaged Notch receptors. Notch receptors on the cell surface may engage with ligand to be activated in trans-, be inhibited in cis- [40], or be endocytosed and turned over without engaging Delta ligand [41]. Previous studies show that Scabrous stabilizes cell surface Notch, and that the binding of Notch with Scabrous precludes binding of Notch with Delta ligand [20, 21]. Together with the findings in our study, we favor a model in which (1) loss of Scabrous expression destabilizes cell surface Notch. In combination with Notch receptor turnover, this state leads to decreased engagement with Delta and lower Notch response in epithelial cells (as in Fig 3B RNAi). (2) Overexpression of Scabrous leads to stabilization of Notch on the cell surface but prevents the ligand from binding to the receptor, also leading to lower Notch response in epithelial cells (as in Fig 3B Sca). The observation that adjacent cells are not as strongly affected as distant cells may be related to the relative affinity of Notch for Delta vs Scabrous. If the relative affinity of Notch for Delta is higher than for Scabrous, and since adjacent epithelial cells have more opportunities to interact with Delta ligand on contact surfaces than distant epithelial cells do via signaling filopodia contacts alone, we would expect to see a higher Notch response in adjacent cells.

## Conclusions

In summary, our evidence demonstrates that Scabrous can be distributed basally via actin-dependent processes during the patterning of the notum epithelium. Consistent with previous research, Scabrous is important for enhancing Notch signaling during lateral inhibition. Our analysis at the level of individual cells clarifies earlier observations that decreasing and increasing levels of Scabrous both result in similar tissue patterning defects. Future work will be needed to address the detailed mechanism by which Scabrous modulates Notch signaling during bristle patterning, and how its signal is transmitted via signaling filopodia.

## Materials and methods

### Fly husbandry

Drosophila stocks were maintained on standard Drosophila food (JazzMix, Fisher Scientific), at 18°C with 24 hour light cycle. Crosses are maintained at room temperature (23–25°C). White pre-pupae were screened against balancers and then aged at 18°C for 24 hours in humidified chambers. Pupae aged to ~12–14 hours after pupariation were then dissected for live or fixed imaging protocols.

### Immunofluorescence

Dissected nota of ~12–14 hAP pupae were fixed for 20 minutes in 4% paraformaldehyde/1X PBS solution. Tissues were then blocked in 1:1 blocking buffer (5% w/v BSA, 3% FBS in 1X PBS) in 1X PBST at room temperature for 1 hour. Tissues were then incubated in primary antibody (see reagents table) with 5% blocking buffer in 1X PBST for 2 hours at room temperature or 4°C overnight. Tissues were washed twice in 1X PBST for 10 minutes each at room temperature, followed by incubation with secondary antibody (see reagents table) in 1X PBST containing fluorescently-labeled phalloidin and DAPI, for 2 hours at room temperature or 4°C overnight. Tissues were washed twice in 1X PBST for 10 minutes each, and then equilibrated in 50% glycerol overnight. Nota were then mounted on coverslips and sealed with nail varnish for storage at 4°C until imaged.

For experiments that distinguish extracellular and total Scabrous protein, we dissected and fixed tissues as stated above. Tissues were first blocked in 1:1 blocking buffer in 1X PBS, then incubated with mouse anti-scabrous for 1 hour at room temperture (5% blocking buffer in 1xPBS). Tissues were washed twice in 1X PBS for 10 minutes each, followed by incubation with TRITC labeled anti-mouse secondary in 1X PBS for 1 hour at room temperature. After two additional washes in 1X PBS, 1X PBST was added for 10 minutes to permeabilize the tissues. Tissues were then treated by standard immunofluorescence protocol outlined above, starting with block (1:1 blocking buffer in 1X PBST) this time using AlexaFluor 647 labeled anti-mouse secondary to label total Scabrous protein.

### Pharmacological treatments

Cytochalasin B stock solutions were made with DMSO and aliquots maintained at -20°C. For experimental treatments, Cytochalasin was diluted to 20ug/mL in room temperature modified Clone 8 medium (2.5% Insect media supplement, 2% FBS, in 1X Schneider’s Medium) [29]. The same volume of DMSO was added to medium for control treatments. The genotype used for this experiment is neur-GAL4, UAS-GMCA/+. Dissected nota were incubated at room temperature, in a humidified chamber, with either 20ug/mL cytochalasin B or control solution for the time points described in Fig 2. At the end of the incubation period, the solution was removed and fixative was added, followed by immunofluorescence protocols described above. For live imaging, fully dissected nota were held in place on a Matek dish by the fibrin clot method [42] in a small volume of medium. An equal volume of modified Clone 8 medium containing vehicle or cytochalasin was added, to reach the appropriate final concentration.

### Imaging

Samples were imaged on either a Leica DMi8 SPE confocal microscope using LASX software, or Nikon C2+ confocal microscope using NIS Elements. The pupal cases of live ~12–14 hAP pupae were removed, exposing the head and thorax. A coverslip coated with a thin film of Halocarbon 27 oil (Sigma) was placed on top of spacers such that only the dorsal thorax contacted the coverslip (as previously published [29]). Live pupae were imaged using a x40 (0.8 NA) air objective. Fixed tissues were imaged using a x40 (1.15NA), x63 (1.3 NA) oil objectives or Nikon C2+ using a x40 (1.3 NA). Ex vivo pharmacological experiments were performed at room temperature on a Nikon EclipseTi, x60 (1.4 NA).

### Quantification and statistical analysis

All image analysis was performed in FIJI/ImageJ. Fig 1B: apical and basal distribution of Scabrous was determined by analyzing z-slices apical or basal to the cell nucleus marked by anti-senseless. Fig 1C: Area adjusted number of Scabrous puncta was calculated by the equation f(x) = x / ((d+1)^2^-d^2^). Fig 2E: A z-projection including basal planes of the bristle precursor cell were generated to visualize the entire length of filopodia present. Lengths were measured manually using FIJI/ImageJ from filopodia tip, along the filopodia length, to the base (intersection with the cell body). Fig 2F–2G: A circular ROI with radius of 15 μm was centered on a bristle precursor labeled with anti-GFP. The number of TRITC +, GFP- puncta within this ROI was counted as local Scabrous and TRITC+, GFP+ puncta were counted as intracellular Scabrous. Apical puncta were distinguished from medial and basal puncta, and only medial and basal puncta were included in our analysis, as they are most likely to be distributed by bristle precursor cell filopodia. Fig 3B: An ROI with a height and width of ~3 μm was centered on the nuclei of NsfGFP expressing epithelial cells (see S3 Fig), and the average fluorescence intensity was measured. Adjacent or distant cell nuclei were determined by relative positioning to the nearest RFP+ nuclei (bristle cells are marked by neur-H2B:mRFP). Fluorescence intensity measurements were taken from a single z-slices at the middle of cells, due to issues with Scabrous-GFP overexpression obstructing nuclear NsfGFP measurements in maximum intensity projections.

Statistical analysis was performed using GraphPad Prism. Specific statistical tests are described in the figure legends.

## Reagents

**Table:** 

Fly stocks	Source	Reference
NsfGFP, neur:H2B-RFP/CyOGFP; pannier-GAL4/TM6BTb	Hunter Lab	[6]
UAS-scabrous RNAi / CyOGFP	BDSC	Stock #63585
UAS-scabrous GFP / CyOGFP	Hunter Lab	Derived from Stock #58459
Scabrous^GSFTF^	BDSC	Stock #64443
UAS-LifeAct-Ruby; pannier-GAL4/TM6BTb	Hunter Lab	Derived from Stock #35545
UAS-LifeAct-Ruby/CyO; neur-GAL4/TM6BTb	Hunter Lab	Derived from Stock #35545
UAS-white RNAi	BDSC	Stock #33644
Neur-GAL4, UAS-GMCA /TM6BTb	Hunter Lab	[14]
W^1118^	BDSC	Stock#3605

**Table:** 

Antibody / Stain	Dilution / Source	Reference
Mouse anti-scabrous	1:500, DSHB	[24]
Mouse anti-Rab7	1:200, DSHB	(Riedel et al., 2016) [27]
Guinea pig anti-senseless	1:2000, Bellen Lab	[43]
Chicken anti-GFP	1:1000, EMD Millipore	AB16901
AF488 anti-chicken	1:2000, Jackson Immunological	703-545-155
555 anti-guinea pig	1:2000, Invitrogen	A21435
TRITC anti-mouse	1:2000, Jackson Immunological	715-025-151
647 anti-mouse	1:2000, Invitrogen	A48289
Phalloidin (555 or 670)	1:500, Cytoskeleton Inc	PHDH1, PHDN1
DAPI	1:1000, Thermoscientific	62248

**Table:** 

Pharmacological reagent	Dilution/Source	Reference
Cytochalasin B	20ug/mL, Tocris	5474
DMSO	Sigma	D5879

## Supporting information

S1 Movie Volume rendering of apicobasal distribution of Scabrous protein in a bristle precursor cell. Green = anti-GFP. Red = anti-Scabrous. Genotype: neur-GAL4, UAS-GMCA/+. Scale bars in microns. Apical plane at (0,0,0).
(AVI)

S1 Fig Apicobasal distribution of Scabrous in notum tissue at ~13 hAP. 0 μm = basal most plane. Each successive panel is +1 μm in the apical direction. Green = phalloiding staining filamentous actin; red = anti-senseless; blue = DAPI staining DNA; white = anti-scabrous. Arrowheads point to first appearance of a new anti-Scabrous puncta. Subsequent appearances of the same puncta are not labeled, for clarity. Higher apparent signal in apical most planes (+5–6) in the anti-Scabrous channel are due to background illumination with 647 nm wavelength laser. Scale bar, 10 μm. Genotype: w^1118^.
(TIF)

S2 Fig Additional analysis of Cytochalasin B treatments. (A) Apical and basal planes for cells in tissues treated as in main Fig 2C–2G, with the addition of the fluorescent phalloidin channel, which stains total filamentous actin (white). Green = anti-GFP, bristle precursor cell; red = anti-Scabrous; blue = DAPI, staining DNA. Tissues have been treated for 5 minutes with either DMSO (upper panels) or Cytochalasin B (lower panels). Apical panels demonstrate that junctional actin is still observable even after 5 minutes of Cytochalasin B treatment. Basal panels demonstrate that basal signaling filopodia are sensitive to 5 minutes of cytochalasin B treatment. Genotype: neur-GAL4, UAS-GMCA/+. (B) Intracellular Scabrous puncta of bristle precursor cells analyzed in main Fig 2F–2G. Anti-Scabrous positive puncta that colocalized with anti-GFP were considered intracellular for bristle precursor cells. Apical puncta were counted in a single apical z-plane. (n) = number of cells analyzed. A minimum of 5 nota were analyzed per condition. NS values: 5 min DMSO v CytB, p>0.99; 30 min DMSO v CytB, p = 0.57; 60 min DMSO v CytB, p>0.46, by Kruskal-Wallis multiple comparisons test. Mean ± SD shown. (C) Basal puncta were counted in a single basal z-plane. Treatment times and conditions are listed. NS values: 5 min DMSO v CytB, p>0.99; 30 min DMSO v CytB, p = 0.39; and *, p = 0.05 by Kruskal-Wallis multiple comparisons test. Mean ± SD shown.
(TIF)

S3 Fig Details of NsfGFP analysis in Fig 3. 12h AP pupae of the genotype NsfGFP, neur-H2B:mRFP/UAS-Scabrous-GFP; pnr-GAL4/+ were imaged for 12 hours to quantify Notch response (nuclear GFP levels). Each panel is a z-plane from one region at 2 μm steps through the tissue. (A) Region of interest from inside the pnr-GAL4 domain, which overexpresses scabrous^GFP^. Bristle precursor cells are labeled by neur-H2B:mRFP (red) nuclei. Example nuclear ROI for measuring NsfGFP is shown in the 4^th^ panel to the right. Nuclear ROI measurements are taken at the z-plane where the nuclei diameter is largest. Note that at this plane, Sca-GFP puncta do not overlap with the nucleus. (B) Region of interest from outside of the pnr-GAL4 domain, in the same pupae as (A). Note the absence of scabrous^GFP^ puncta. Example nuclear ROI for measuring NsfGFP is shown in the 3^rd^ panel to the right. Scale bars, 5 μm.
(TIF)
