# Supplementary figures and images for: Scabrous is distributed via signaling filopodia to modulate Notch response during bristle patterning in Drosophila
Source: PLoS One. 2023 Sep 20;18(9):e0291409. doi: 10.1371/journal.pone.0291409 (PMC10511103; doi:10.1371/journal.pone.0291409)

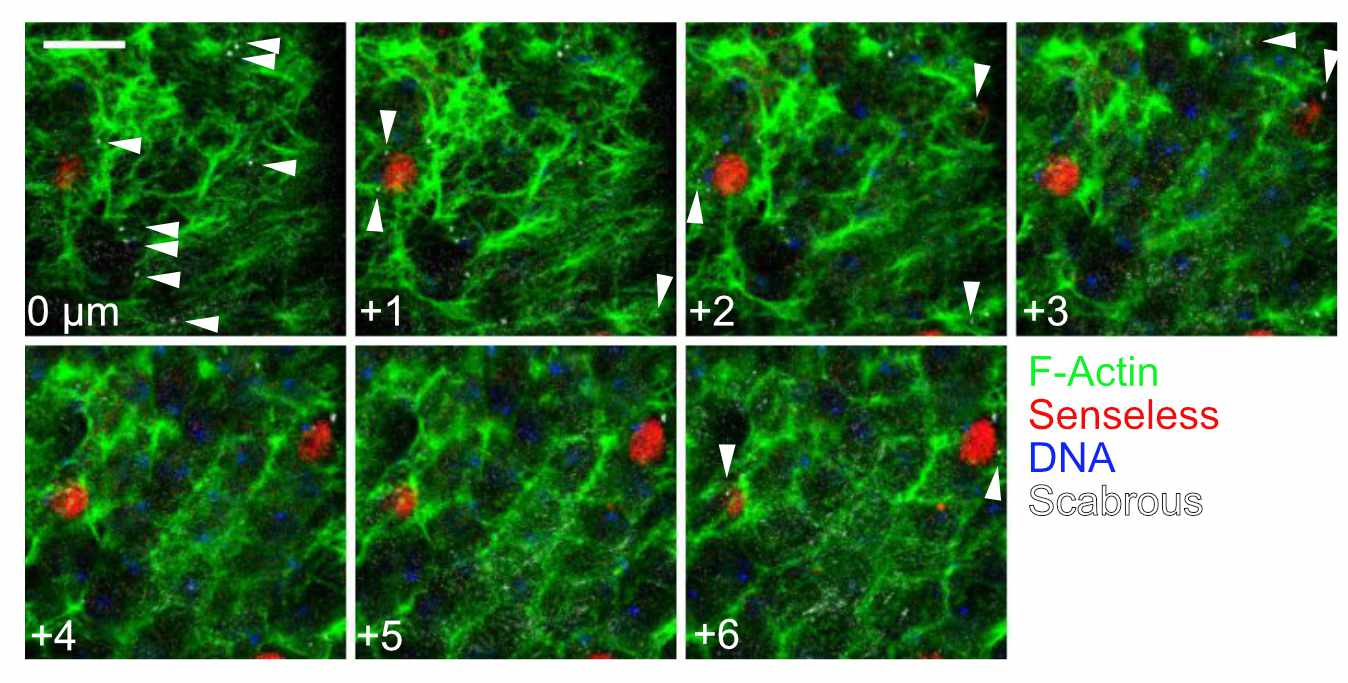

Supplement: S1 Fig — 0 μm = basal most plane. Each successive panel is +1 μm in the apical direction. Green = phalloiding staining filamentous actin; red = anti-senseless; blue = DAPI staining DNA; white = anti-scabrous. Arrowheads point to first appearance of a new anti-Scabrous puncta. Subsequent appearances of the same puncta are not labeled, for clarity. Higher apparent signal in apical most planes (+5–6) in the anti-Scabrous channel are due to background illumination with 647 nm wavelength laser. Scale bar, 10 μm. Genotype: w1118. (TIF) [file pone.0291409.s002.tif]

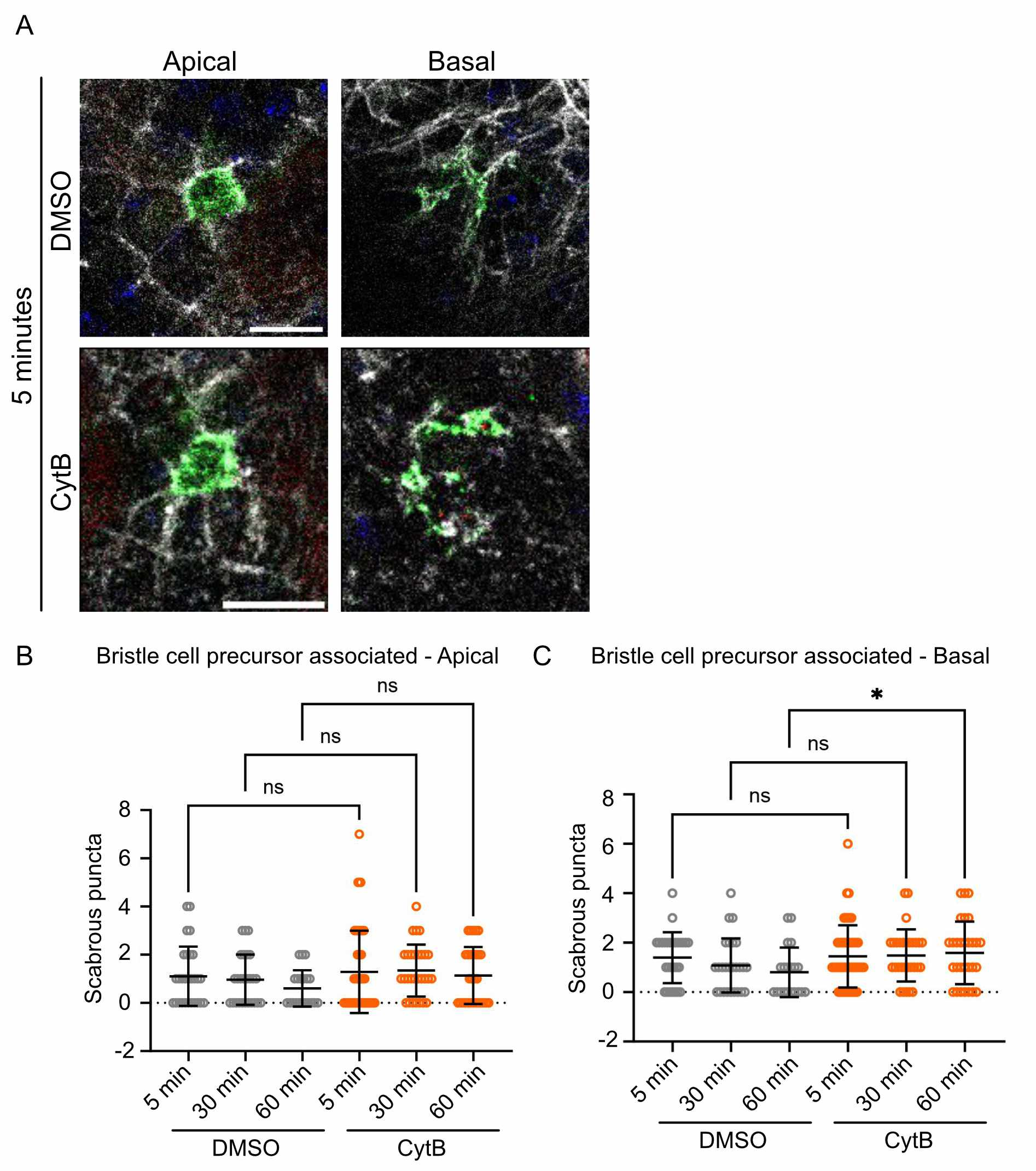

Supplement: S2 Fig — (A) Apical and basal planes for cells in tissues treated as in main Fig 2C–2G, with the addition of the fluorescent phalloidin channel, which stains total filamentous actin (white). Green = anti-GFP, bristle precursor cell; red = anti-Scabrous; blue = DAPI, staining DNA. Tissues have been treated for 5 minutes with either DMSO (upper panels) or Cytochalasin B (lower panels). Apical panels demonstrate that junctional actin is still observable even after 5 minutes of Cytochalasin B treatment. Basal panels demonstrate that basal signaling filopodia are sensitive to 5 minutes of cytochalasin B treatment. Genotype: neur-GAL4, UAS-GMCA/+. (B) Intracellular Scabrous puncta of bristle precursor cells analyzed in main Fig 2F–2G. Anti-Scabrous positive puncta that colocalized with anti-GFP were considered intracellular for bristle precursor cells. Apical puncta were counted in a single apical z-plane. (n) = number of cells analyzed. A minimum of 5 nota were analyzed per condition. NS values: 5 min DMSO v CytB, p>0.99; 30 min DMSO v CytB, p = 0.57; 60 min DMSO v CytB, p>0.46, by Kruskal-Wallis multiple comparisons test. Mean ± SD shown. (C) Basal puncta were counted in a single basal z-plane. Treatment times and conditions are listed. NS values: 5 min DMSO v CytB, p>0.99; 30 min DMSO v CytB, p = 0.39; and *, p = 0.05 by Kruskal-Wallis multiple comparisons test. Mean ± SD shown. (TIF) [file pone.0291409.s003.tif]

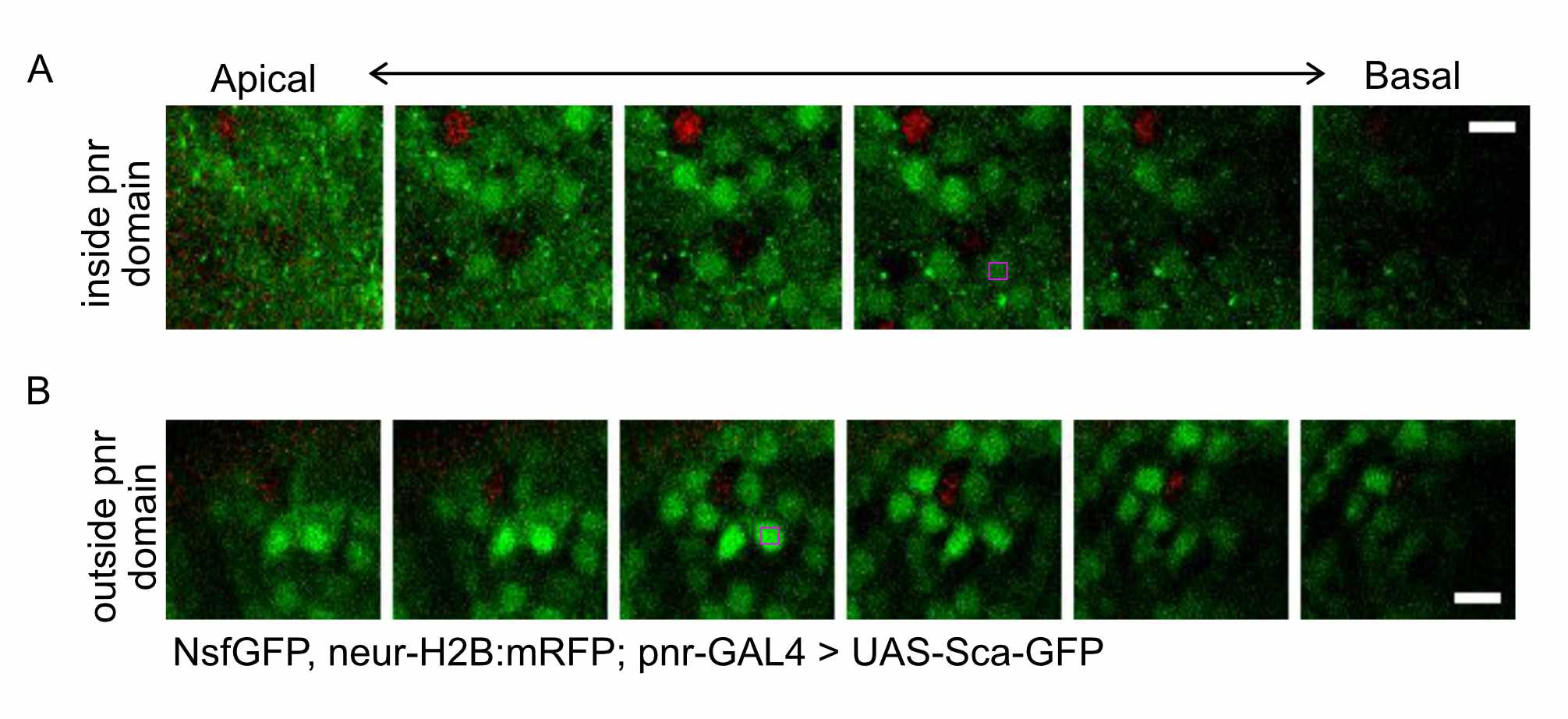

Supplement: S3 Fig — 12h AP pupae of the genotype NsfGFP, neur-H2B:mRFP/UAS-Scabrous-GFP; pnr-GAL4/+ were imaged for 12 hours to quantify Notch response (nuclear GFP levels). Each panel is a z-plane from one region at 2 μm steps through the tissue. (A) Region of interest from inside the pnr-GAL4 domain, which overexpresses scabrousGFP. Bristle precursor cells are labeled by neur-H2B:mRFP (red) nuclei. Example nuclear ROI for measuring NsfGFP is shown in the 4th panel to the right. Nuclear ROI measurements are taken at the z-plane where the nuclei diameter is largest. Note that at this plane, Sca-GFP puncta do not overlap with the nucleus. (B) Region of interest from outside of the pnr-GAL4 domain, in the same pupae as (A). Note the absence of scabrousGFP puncta. Example nuclear ROI for measuring NsfGFP is shown in the 3rd panel to the right. Scale bars, 5 μm. (TIF) [file pone.0291409.s004.tif]
